# Molecular Evolution of the Vertebrate FK506 Binding Protein 25

**DOI:** 10.1155/2014/402603

**Published:** 2014-03-02

**Authors:** Fei Liu, Xiao-Long Wei, Hao Li, Ji-Fu Wei, Yong-Qing Wang, Xiao-Jian Gong

**Affiliations:** ^1^Department of Pharmacology, China Pharmaceutical University, Nanjing 210009, China; ^2^Research Division of Clinical Pharmacology, The First Affiliated Hospital, Nanjing Medical University, 300 Guangzhou Road, Nanjing 210029, China; ^3^Department of Pathology, Cancer Hospital of Shantou University Medical College, Shantou, China

## Abstract

FK506 binding proteins (FKBPs) belong to immunophilins with peptidyl-prolyl isomerases (PPIases) activity. FKBP25 (also known as FKBP3) is one of the nuclear DNA-binding proteins in the FKBPs family, which plays an important role in regulating transcription and chromatin structure. The calculation of nonsynonymous and synonymous substitution rates suggested that FKBP25 undergoes purifying selection throughout the whole vertebrate evolution. Moreover, the result of site-specific tests showed that no sites were detected under positive selection. Only one PPIase domain was detected by searching FKBP25 sequences at Pfam and SMART domain databases. Mammalian FKBP25 possess exon-intron conservation, although conservation in the whole vertebrate lineage is incomplete. The result of this study suggests that the purifying selection triggers FKBP25 evolutionary history, which allows us to discover the complete role of the PPIase domain in the interaction between FKBP25 and nuclear proteins. Moreover, intron alterations during FKBP25 evolution that regulate gene splicing may be involved in the purifying selection.

## 1. Introduction

Immunophilins include three families with peptidyl-prolyl isomerases (PPIases) activity, FK506 binding proteins (FKBPs), cyclophilins, and parvulins. FKBPs are named for binding to the immunosuppressive drug FK506, characterized by one or more PPIase domains. The 15 identified members of human FKBPs are divided into 4 groups: cytoplasmic, TPR domain, endoplasmic reticulum (ER), and nucleus. FKBP25 and FKBP133 locate in the nucleus, containing a single PPIase domain [1].

FKBP25 (also known as FKBP3) is the first mammalian FKBP with a calculated molecular mass of 25 kDa found in the nucleus, which plays a role in regulating transcription and chromatin structure. The FKBP25 comprises a conserved PPIase domain at its C-terminus with a 43% sequence identity to FKBP12 and a helix-loop-helix (HLH) motif at its unique hydrophilic N-terminal [2, 3]. This conserved PPIase domain functions in binding to the immunosuppressive agent FK506 or rapamycin. Unlike another FKBPs, FKBP25 shows a strong affinity for binding rapamycin (Ki = 0.9 nM) over FK506 (Ki = 200 nM) [4]. The FKBP25 was reported to be associated with nuclear proteins including transcription factor Yin-Yang1 (YY1), mouse double minute 2 (MDM2), and histone deacetylases (HDACs) [5]. FKBP25 binds to YY1 at N-terminal and increases its DNA-binding activity without the involvement of the FK506/rapamycin binding domain [6]. In addition, the level and activity of the tumor suppressor protein p53 are negatively regulated by MDM2. The HLH motif of FKBP25 mediates protein-protein interaction to enhance ubiquitination and degradation of oncogene MDM2, increasing the expression of tumor suppressor p53 and its downstream effector p21 [7]. Moreover, the protein-protein interaction contributes to form HDAC complexes, which is critical for the chromatin structure [2].

In 1992, Jin et al. reported the molecule cloning of human FKBP25 and performed a homology comparison between FKBP25 and FKBP12/FKBP13 [8]. Furthermore, Mas et al. showed the molecule cloning of mouse FKBP25 and expression pattern of FKBP25 gene during cerebral cortical neurogenesis [9]. However, the relationships between nuclear functions and evolution in FKBP25 are seldom reported. In this study, we exhibit an evolutional analysis not only on selective pressure but also on intron-exon conversion among vertebrate FKBP25 genes.

## 2. Materials and Methods

### 2.1. Sequence Data Collection

All the FKBP25 gene and amino acid sequences were obtained from the ENSEMBL (http://www.ensembl.org/index.html) [10], based on orthologous and paralogous relationships. The gained FKBP25 sequences were applied as queries to search known FKBP25 genes using BLAST at the National Center for Biotechnology Information (NCBI), in order to confirm whether their best hit was an FKBP25 gene [11].

Incomplete sequences of FKBP25 genes in four species (tree shrew, horse, platypus, and turkey) were retrieved from both ENSEMBL and NCBI. After eliminating these incomplete sequences, 28 sequences were applied for this study. The 28 sequences from 23 species comprised *human* (ENSG00000100442), *chimpanzee* (ENSPTRG00000006305), *gorilla* (ENSGGOG00000013322), *orangutan* (ENSPPYG00000005778), *macaque* (ENSMMUG00000016512), *marmoset *(ENSCJAG00000015972), *mouse* (ENSMUSG00000020949), *rat* (ENSRNOG00000004629), *guinea pig* (ENSCPOG00000001444), *rabbit1* (ENSOCUG00000007535), *rabbit2* (ENSOCUG00000026892), *dog1* (ENSCAFG00000014018), *dog2* (ENSCAFG00000014093), *dog3 *(ENSCAFG00000024192), *dog4* (ENSCAFG00000000578), *cow *(ENSBTAG00000002610), *elephant1* (ENSLAFG00000003572), *elephant2* (ENSLAFG00000027553), *opossum* (ENSMODG00000007352), *chicken* (ENSGALG00000012466), *zebra finch* (ENSTGUG00000013231), *anole lizard* (ENSACAG00000004080), *xenopus* (ENSXETG00000003052), *fugu* (ENSTRUG00000011887), *medaka* (ENSORLG00000015070), *stickleback* (ENSGACG00000012834), *tetraodon* (ENSTNIG00000010980), and *zebrafish *(ENSDARG00000079018).

### 2.2. Molecular Phylogenetic Analyses

The protein coding sequences of FKBP25 were aligned using CLUSTAL W program in MEGA 5.05. We constructed a maximum likelihood (ML) tree of FKBP25 amino acid sequences by MEGA 5.05 with the optimal model (Kimura 2-parameter model). The relative support of internal node was performed by bootstrap analyses with 1000 replications for ML reconstructions [12].

### 2.3. Selection Pressure Analyses

The numbers of nonsynonymous substitutions per nonsynonymous site (*dN*) and the numbers of synonymous substitutions per synonymous site (*dS*) were computed by MEGA 5.05 with the modified Nei-Gojobori method. The *dN/dS* <1, =1 and >1 demonstrate purifying selection, neutral selection, and positive selection, respectively [13]. The *dN* is the numbers of nonsynonymous substitutions per nonsynonymous site, and the *dS* is the numbers of synonymous substitutions per synonymous site. The transition/transversion ratio was 1.55 estimated using the ML method by MEGA 5.05 [14].

The FASTA format of FKBP25 sequences was converted to the PAML format using DAMBE software for subsequent site analyses [13]. The CODEML program implemented in the PAML 4.7 package was used to detect positive selection of individual sites. The site-specific model was exerted using likelihood ratio tests (LRT) to compare M7 (null model) with M8 model. M7 is a null model that does not allow for any codons with *ω* > 1, whereas M8 model allows for positively selective sites (*ω* > 1). When the M8 model fitted the data significantly (*P*-value < 0.05) better than the null model (M7), the presence of sites with *ω* > 1 is suggested. On the contrary, the results of *P* value > 0.05 proved the absence of sites with *ω* > 1. The twice log likelihood difference between the two compared models (2Δ*l*) is compared against *χ* ^2^ with critical values 5.99 and 9.21 at 0.05 and 0.01 significance levels, respectively [15].

### 2.4. Protein Domain and Motif Analyses

Protein domain analyses of FKBP25 were shown at Pfam domains database (http://pfam.sanger.ac.uk) [16]. SMART (http://smart.embl-heidelberg.de/) was used to make sure the presence of FKBP25 domains [17]. The motifs of FKBP25 were analyzed by the MEME software (http://meme.sdsc.edu/meme/website/intro.html) with a maximum of 10 motifs to find [18].

### 2.5. Exon-Intron Conservation Analyses

We collected elaborate information about FKBP25 exon and intron from ENSEMBL (http://www.ensembl.org/index.html) [19]. The number and length of FKBP25 exon and intron in 28 sequences were investigated for exon-intron conservation analyses.

## 3. Results

### 3.1. Phylogenetic Analyses of FKBP25

**Figure 1:**
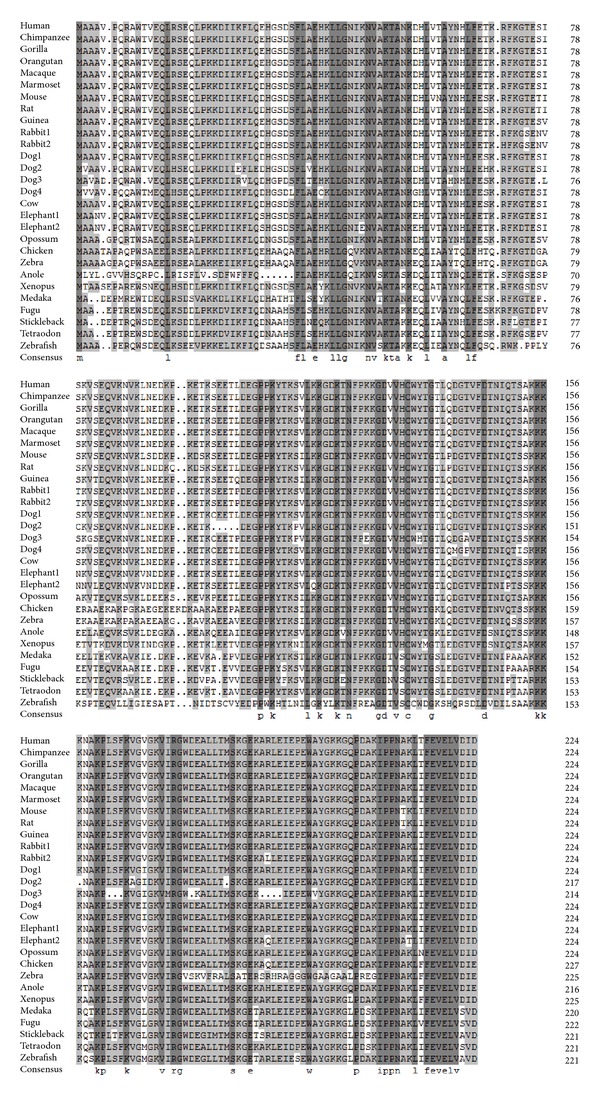
Sequence and structural alignment of FKBP25.

**Figure 2:**
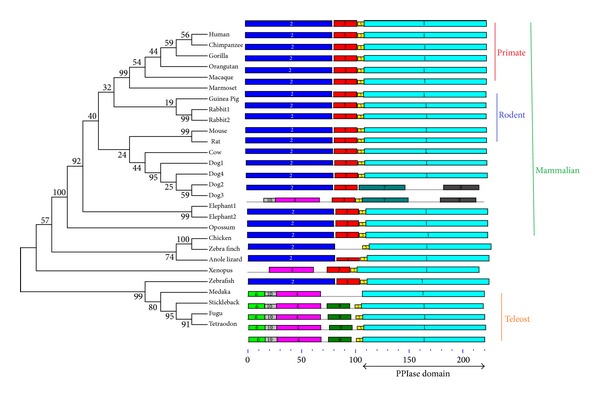
Phylogenetic tree and motif distributions of FKBP25.

All the FKBP25 gene and protein sequences were collected from the ENSEMBL and checked by BLAST at NCBI. The sequence and structural alignment of FKBP25 was shown in Figure 1. The phylogenetic tree was constructed according to the protein coding sequences of FKBP25 using the maximum likelihood method (Figure 2, left panel). The FKBP25 genes from the primate lineage and teleost lineage form a species-specific cluster, respectively. Four FKBP25 isoforms of *dog* exhibited a close relationship and clustered together, according to the phylogenetic tree. There were similar phenomena in *rabbit* and *elephant*.

### 3.2. Selection Pressure Analyses

**Figure 3:**
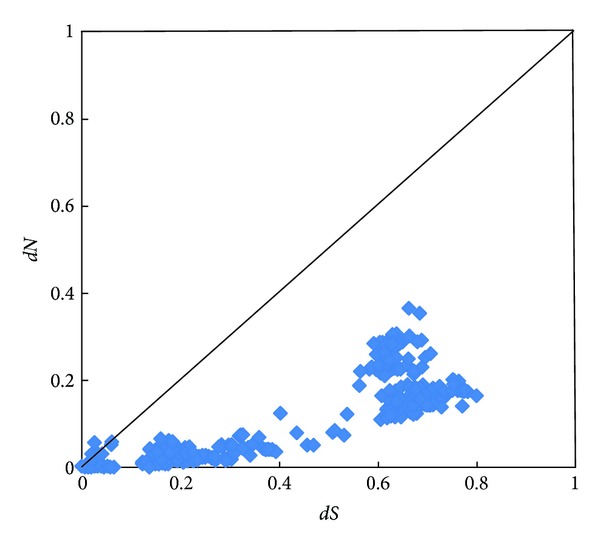
Pairwise comparisons of *dN* and *dS* among 28 vertebrate FKBP25 sequences.

**Figure 4:**
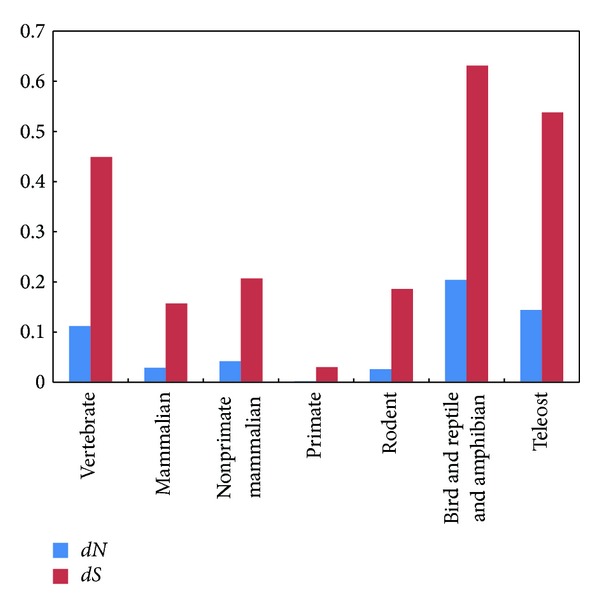
The average nonsynonymous (*dN*) and synonymous (*dS*) in FKBP25 from different vertebrate groups. The value of average *dN* was in blue, and the value of average *dS* was in red.

The nonsynonymous to synonymous rate ratio (*dN/dS*) may demonstrate the selective pressures of involved protein. We calculated the pairwise distance of FKBP25 sequences using MEGA 5.05. There was a significantly lower *dN* than *dS* in the pairwise comparisons of these sequences. Most values of *dN/dS* in these sequences were distributed blow the diagonal, showing that the presence of a purifying selection existed in the FKBP25 (Figure 3). The comparisons of average *dN* and *dS* in various vertebrate groups were shown in Figure 4, respectively. Furthermore, site-specific tests were performed for searching the positive selection sites in vertebrate, mammalian, primate, and mammalian excluding primate, rodent and teleost lineages. Although some positive selection sites were computed, each 2Δ*l* of M7 and M8 <5.99 indicated that the M8 model was not significantly better than the M7 model to fit the data. Consequently, we concluded that the site-specific analyses also compute no positive selection sites acting on FKBP25 using PAML4.7 (Table 1).

### 3.3. Protein Domain and Motif Analyses

**Figure 5:**
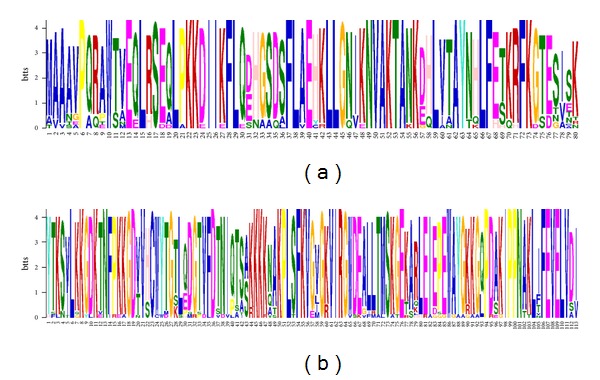
Sequence logos (MEME LOGOs) of conserved motifs identified in vertebrate FKBP25.

Early studies reported that mammalian FKBP25 have two portions: one is a putative helix-loop-helix motif within N-terminal unique sequence (Figure 5(a)) and the other is the PPIase domain at its C-terminus (Figure 5(b)) [20].

The domain distribution of FKBP25 was investigated using FKBP25 to search amino acid sequences at the Pfam database firstly. Only one domain (PPIase domain) was found in the Pfam database. The PPIase domain within FKBP25 sequences generally started at position 122 and ended at position 221. Similarly, we further make sure that the FKBP25 domain is at SMART, resulting in the single PPIase domain at position 119 to 221.

We then performed a detailed domain and motif analyses using the MEME software. Except two *dog* isoforms, *dog2* and *dog3*, the FKBP25 sequences used in this study contain a conversed PPIase domain within motif 1 (shown in Figure 2) at its C-terminus. In addition, the result implied that motif 2 located in the N-terminal contained an HLH motif [6], which was associated with DNA binding and dimerization [21]. However, HLH motif was not found in *dog3*, *anole lizard,* and *teleost* lineage, implying that these FKBP25 proteins may function on gene expression in another pathway.

### 3.4. Exon-Intron Conservation Analyses

**Figure 6:**
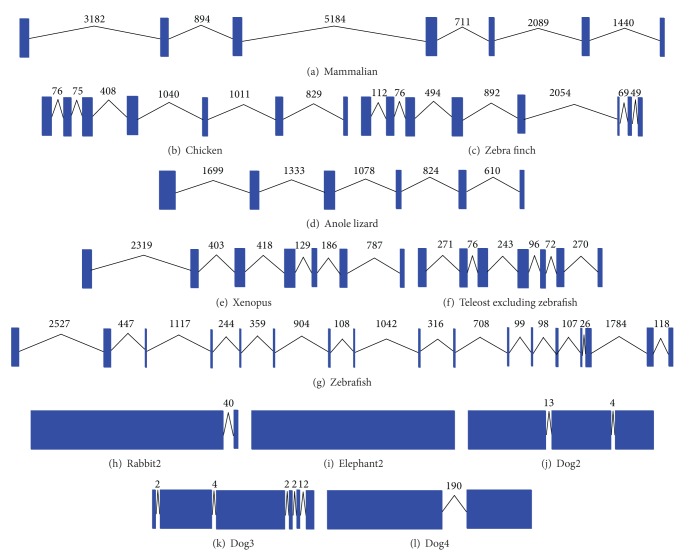
Exon-intron conservation among FKBP25 genes.

The exon-intron information collected from the ENSEMBL database was shown in Table 2 and Figure 6. Most of the FKBP25 genes have 7 exons with similar length in different species (Table 2). Mammalian FKBP25 shows exon-intron conservation with 6 introns and similar sizes of each intron. Intron deletions existed in several isoforms of species. The *rabbit2* isoform had 2 exons, and *elephant2* isoform had only one exon. The exon numbers of *dog2, dog3,* and *dog4* isoforms were less than seven. Except mammalian FKBP25 genes, *anole lizard* reduced one exon compared with mammalian and birds, but the *xenopus* and teleost maintained 7 exons. The intron deletions of FKBP25 genes may happen in the evolutionary process from amphibian to reptile. Then, a subsequent intron insertion occurred in the evolution from reptile to more advanced animals. The FKBP25 genes also had intron insertion in *zebra finch* and *zebra fish.*

## 4. Discussion

FKBP25 is a nuclear member of the FKBPs family that is associated with transcription and chromatin structure [2]. The interactions of FKBP25 with nuclear proteins are closely associated with HLH motif at the N-terminal of FKBP25. However, whether the PPIase domain at C-terminus is important for these interactions remains uncertain. The selection pressure analyses revealed that the purifying selection triggered a whole evolutionary history of FKBP25 in vertebrates, even in each lineage of vertebrates. Purifying selection is one of the natural selections that resist deleterious mutations with negative selective coefficients [22]. The mutations that disrupt the correct folding of the FKBP25 domain can weaken PPIase activity and may be the deleterious mutations [5]. It was hypothesized that the mutations of PPIase domain were one of explanations behind the purifying selection throughout FKBP25 evolution. Therefore, although the PPIase domain of FKBP25 was not found to be involved in the protein interactions previously, the PPIase domain might have some associations with the YY1 DNA-binding, MDM2 autoubiquitination and degradation, and HDACs complex formation. These inferences will become a potent direction for exploring the relationship between nuclear proteins and PPIase domain in the future.

The protein-coding sequence length of vertebrate FKBP25 is highly conversed that almost all the taxa are 224 bp; nevertheless the original gene length and exon-intron status are tremendously various among vertebrate species. However, mammalian FKBP25 exhibit exon-intron conservation with 6 introns and similar sizes of each intron. *Chicken* FKBP25 maintains 6 introns, but *zebra finch* has one more intron that inserts in the gene. Similarly, a large variability of intron number and sizes among all the taxa shown in Figure 6 revealed that intron insertion and deletion events happened frequently during the FKBP25 evolutionary history from teleost to birds. In particular, *zebrafish* demonstrated the maximum number of introns in this study, and the size of exon is much smaller than other teleost species (Figure 6(g)). The intron loss of FKBP25 gene from species more advanced than *zebrafish* is likely to induce alterations of gene expression due to the absence of specific intron splicing. Under the purifying selection, the FKBP25 gene expression event continuously removes the pernicious mutations that may associate with intron splicing regulation [23].

FKBP25 gene knockdown declined the expression levels of p53 and p21, which emphasized the significance of FKBP25 in regulating p53 and subsequently p21 expression through controlling the ubiquitination of MDM2. Both the FKBP25 PPIase domain and its N-terminal portion were critical for the ubiquitination and degradation of MDM2 [2]. Moreover, Jin et al. reported that FKBP25 prefers to bind to rapamycin rather than FK506, implying that FKBP25 may be an important target molecule for immunosuppression by rapamycin [8]. All the evolution analyses indicated the conservation of FKBP25 gene in vertebrates. Therefore, FKBP25 possesses some basic functions in vertebrate species, like regulating p53 and p21 expression and binding to rapamycin for immunosuppression, reinforcing the suggestion that the purifying selection triggered the evolution of vertebrate FKBP25.

In conclusion, FKBP25 as a nuclear FKBP subjects to the purifying selection throughout the whole evolution, which implied the complete role of the PPIase domain involved in the interaction between FKBP25 and the nuclear proteins that are needed to be discovered continually. Additionally, incomplete exon-intron conservation of FKBP25 meets the vertebrate lineage. The intron gain or loss among the taxa is likely to be involved in the purifying selection.

## Figures and Tables

**Table 1:** Site-specific tests for positive selectionof FKBP25.

Species	Models	Estimates of parameters	lnL	2Δ*l*	Positively selected sites
Vertebrate	M7	*p* = 0.91900 *q* = 8.19764	−5463.938465	0.003806	NA
	M8	*p*0 = 0.99999 *p* = 0.91899 *q* = 8.19758	−5463.940368		None
		(*p*1 = 0.00001) *w* = 1.86072			

Mammalian	M7	*p* = 0.33823 *q* = 1.62046	−2182.244789	0.000258	NA
	M8	*p*0 = 0.99999 *p* = 0.33824 *q* = 1.62055	−2182.244918		None
		(*p*1 = 0.00001) *w* = 1.00000			

Primate	M7	*p* = 4.13016 *q* = 99.00000	−997.077389	0.000102	NA
	M8	*p*0 = 0.99999 *p* = 4.12942 *q* = 99.00000	−997.077440		None
		(*p*1 = 0.00001) *w* = 1.00000			

Mammalian excluding primate	M7	*p* = 0.28229 *q* = 1.41420	−2242.306222	0.000160	NA
	M8	*p*0 = 0.99999 *p* = 0.28230 *q* = 1.41430	−2242.306302		NS
		(*p*1 = 0.00001) *w* = 1.00000			

Rodent	M7	*p* = 0.13287 *q* = 1.19752	−1372.902164	0.000058	NA
	M8	*p*0 = 0.99999 *p* = 0.13287 *q* = 1.19764	−1372.902193		NS
		(*p*1 = 0.00001) *w* = 1.00000			

Teleost	M7	*p* = 0.38691 *q* = 4.30540	−2354.923181	0.000408	NA
	M8	*p*0 = 0.99999 *p* = 0.38690 *q* = 4.30545	−2354.923385		NS
		(*p*1 = 0.00001) *w* = 3.90806			

lnL: the log-likelihood difference between the two models; 2Δ*l*: twice the log-likelihood difference between the two models (In all the species, 2Δ*l* < 9.21, the *P*-value is more than the significance level 0.05, indicating that M8 model is not better than M7 model); NA: not allowed; NS: not shown (it means the sites under positive selection but not reaching the significance level of 0.9).

**Table 2:** Exon and intron lengths of FKBP25.

Species	Length (bp)															
	Exon1	Intron1	Exon2	Intron2	Exon3	Intron3	Exon4	Intron4	Exon5	Intron5	Exon6	Intron6	Exon7	Intron7	Exon8	Total exons
*Human *	108	3548	102	797	108	8173	136	530	68	2761	98	1775	55	—	—	675
*Chimpanzee *	108	3524	102	797	108	8898	136	530	68	2725	98	1789	55	—	—	675
*Gorilla *	108	3538	102	796	108	8214	136	530	68	2753	98	1778	55	—	—	675
*Orangutan *	108	3498	102	793	108	8395	136	533	68	2457	98	1432	55	—	—	675
*Macaque *	108	3496	102	786	108	8273	136	531	68	2845	98	1818	55	—	—	675
*Marmoset *	108	3592	102	780	108	5644	136	507	68	2537	98	2100	55	—	—	675
*Mouse *	108	3762	102	841	108	2224	136	837	68	1961	98	937	55	—	—	675
*Rat *	108	3528	102	816	108	2030	136	942	68	1667	98	1118	55	—	—	675
*Guinea pig *	108	3232	102	772	108	3600	136	1416	68	1346	98	1340	55	—	—	675
*Rabbit1 *	108	2189	102	1082	108	4634	136	1115	68	1826	98	1266	55	—	—	675
*Rabbit2 *	620	40	55	—	—	—	—	—	—	—	—	—	—	—	—	675
*Dog1 *	108	2573	102	1076	108	2088	136	468	68	1823	98	1216	55	—	—	675
*Dog2 *	296	13	229	4	129	—	—	—	—	—	—	—	—	—	—	654
*Dog3 *	30	2	195	4	252	2	33	2	33	12	102	—	—	—	—	645
*Dog4 *	427	190	248	—	—	—	—	—	—	—	—	—	—	—	—	675
*Cow *	108	2332	102	603	108	2835	136	484	68	1706	98	1309	55	—	—	675
*Elephant1 *	108	3176	102	1089	108	4756	136	483	68	1580	98	1725	55	—	—	675
*Elephant2 *	675	—	—	—	—	—	—	—	—	—	—	—	—	—	—	675
*Opossum *	108	2560	102	1484	108	2807	136	1051	68	1261	98	554	55	—	—	675
*Chicken *	111	76	102	75	114	408	136	1040	68	1011	98	829	55	—	—	684
*Zebra finch *	111	112	102	76	108	494	136	892	97	2054	16	69	53	49	55	678
*Anole lizard *	186	1699	108	1333	136	1078	68	824	98	610	55	—	—	—	—	651
*Xenopus *	111	2319	102	403	108	418	136	129	68	186	98	787	55	—	—	678
*Fugu *	105	375	102	78	105	65	136	82	68	68	98	106	55	—	—	669
*Medaka *	105	109	102	71	99	738	136	75	68	70	98	804	55	—	—	663
*Stickleback *	105	294	102	76	102	93	136	135	68	81	98	96	55	—	—	666
*Tetraodon *	105	305	102	80	102	75	136	91	68	70	98	75	55	—	—	666
*Zebra fish *	105	2527	102	447	16	1117	20	244	16	359	19	904	28	108	11	666

	Intron8	Exon9	Intron9	Exon10	Intron10	Exon11	Intron11	Exon12	Intron12	Exon13	Intron13	Exon14	Intron14	Exon15	Intron15	

	1042	24	316	26	708	14	99	10	98	31	107	15	26	76	1784	

	Exon16	Intron16	Exon17													

	98	118	55													
